# Associations of Intrauterine and Postnatal Weight and Length Gains With Adolescent Body Composition: Prospective Birth Cohort Study From Brazil

**DOI:** 10.1016/j.jadohealth.2012.08.013

**Published:** 2012-12

**Authors:** Jonathan C.K. Wells, Samuel C. Dumith, Ulf Ekelund, Felipe F. Reichert, Ana M.B. Menezes, Cesar G. Victora, Pedro C. Hallal

**Affiliations:** a Childhood Nutrition Research Centre, UCL Institute of Child Health, London, United Kingdom; b Programa de Pós-graduacção em Epidemiologia, Universidade Federal de Pelotas, Pelotas, RS, Brazil; c Department of Sport Medicine, Norwegian School of Sport Medicine, Oslo, Norway; d Escola Superior de Educação Física, Universidade Federal de Pelotas, Rua Luiz de Camões 625, Pelotas, Brazil

**Keywords:** Body composition, Growth, Obesity, Nutritional programming

## Abstract

**Purpose:**

Early growth patterns have been associated with subsequent obesity risk. However, findings from middle-income populations suggest that early infant growth may benefit lean mass and height rather than adiposity. We tested the hypothesis that rapid weight or length gain in different growth periods would be associated with size and body composition in adolescence, in a prospective birth cohort from southern Brazil.

**Methods:**

Body composition was assessed in 425 adolescents (52.2% male) at 14 years. Exposures were birth weight z-score and conditional growth in weight or length for the periods 0–6, 6–12 and 12–48 months. Differences in anthropometric and body composition outcomes between tertiles of growth in each period were tested by one-way analysis of variance.

**Results:**

Size at birth and conditional weight and length at 6 months were associated with later height. The effect of infant weight gain on lean mass was greater for males than females, and effect on fat mass greater for females than males. By early childhood, rapid weight gain generated relatively similar effects on both tissue masses in both sexes. Rapid length gain had stronger effects on outcomes in males than females at each time point, and benefited lean mass more than adiposity. All effects were substantially attenuated after adjusting for current height. Early weight gain was more important than length gain at influencing body composition outcomes in adolescence.

**Conclusions:**

Rapid infant weight and length gains were primarily associated with larger size in adolescence rather than increased adiposity. From one year onwards, associations between rapid weight gain and fat and lean masses remained after adjustment for height.

**Implications and Contribution**

Rapid weight gain in fetal life and the first 6 months of infancy was associated with greater body size, whereas rapid weight gain from one year was associated with greater adiposity, adjusting for height. Rapid early infant weight gain does not increase obesity risk in Brazilian adolescents.

Rapid weight gain in fetal life, infancy, and early childhood has been associated in many studies with the risk of obesity in childhood or adulthood [1–6]. This has led to debate concerning the merits of rapid infant weight gain [7], which often follows a reduced rate of fetal weight gain. In industrialized populations, studies have shown that upward centile-crossing during infancy and childhood is associated with greater whole-body and central adiposity in later life. Infancy has therefore been suggested as an important period of the life-course for obesity prevention, and some have recommended slower weight gain to reduce obesity risk and cardiovascular risk [4,8,9].

Studies from modernizing populations provide conflicting evidence. For example, studies from Guatemala, India, and Brazil have reported that rapid infant weight and length gain are positively associated with subsequent height and lean mass rather than adiposity [10–13]. Associations of weight gain and total or central adiposity appear to strengthen from early childhood onwards [10–13]. However, these studies were in most cases based on body composition values estimated from anthropometry or prediction methods such as bio-electrical impedance analysis. There is therefore a need to confirm these earlier studies using more accurate methodologies.

In Brazil, we previously studied a subset of 172 males from the 1993 prospective Pelotas birth cohort at 9 years. Using bio-electrical impedance to assess lean and fat masses, we found that birth weight and early infant weight gain were positively associated with later height and lean mass, but not with later adiposity [12]. In contrast, greater weight gain from one year onwards was positively associated with greater lean and fat masses, but not with height. These data suggest a developmental shift in the target of weight gain from height and lean mass towards fat mass.

Here, we report a further follow-up of a larger subsample of the same cohort, this time including females as well as males. We studied body composition using isotope dilution in order to increase the accuracy of the measurements. We tested the hypothesis that rapid weight or length/height gain would be associated with each of lean and fat masses in adolescence. As slower weight gain in early life has been recommended by some for cardiovascular health [4,7–9], we compared outcomes between the fastest and slowest tertiles of both weight gain and length gain. We also adjusted weight gain for length gain and vice versa, to clarify which component of growth is most important for the development of body composition.

## Methods

### Subjects

The 1993 Pelotas (Brazil) birth cohort study recruited 5,249 individuals [14]. Anthropometric data were collected in a subsample of 1,272 at 6, 12 and 48 months. For this study, we randomly selected 13% of those born in each calendar month of the year. This identified 655 individuals at one month of age, of whom 453 with full data at previous time points were successfully located and studied in adolescence. These individuals underwent measurements of body composition by deuterium dilution, physical activity by accelerometry and blood pressure at 13.3 years [15]. Ethical approval was obtained from the Federal University of Pelotas Medical School Ethics Committee.

### Anthropometry

Birth weight and length (considered infant equivalent to height) were measured at the hospital by the research team. Weight and length/height at 6, 12 and 48 months were measured at the cohort participant's household. At the 14-year visit, weight and height were again measured. Skinfolds were measured in triplicate using Holtain calipers. Waist was measured at the level of the umbilicus with a nonstretchable tape.

### Body composition

Body composition in adolescence was measured using deuterium [16]. Briefly, each adolescent was given a drink containing approximately .05 g 99.9% deuterium oxide (^2^H_2_O) per kg weight. Saliva samples were obtained pre-dose and 4-hour post-dose using absorbent salivettes at least 30 minutes after the last ingestion of food or drink, and then stored frozen at −30°C, as described in detail elsewhere [15]. The samples were shipped to the UK for analysis in duplicate by mass spectrometry, using the equilibration method (Delta plus XP, Thermofisher Scientific, Bremen, Germany). For calculating total body water (TBW), it was assumed that ^2^H_2_O dilution space overestimated TBW by a factor of 1.044 [17]. Correction was made for dilution of the dose by water intake during the 4-hour equilibration period [16]. Values for TBW were converted to lean mass (LM, used here synonymously with fat-free mass), using new reference data for the hydration of lean tissue [18]. Fat mass (FM) was calculated as the difference of LM and WT. Both FM and LM were then adjusted for height to give the fat mass index (FMI) and lean mass index (LMI), both expressed in the same kg/m^2^ units as BMI [19,20].

Preliminary analyses involved log-log regression analyses, using natural log-transformed data for height (HT), LM, and FM. Regression of LnFM or LnLM on LnHT produced coefficients for LnHT of 2.7 (SE 1) in males and 1.5 (SE .8) in females for LnLM, and 4.1 (SE 1) in males and 2.6 (SE .2) in females for LnFM. These represent relevant values of n, whereby an index M/HT^n^ adjusts M (an index of weight) for HT. Although two of these values were significantly different from 2, correlations of FMI or LMI and height remained <.25, indicating that these outcomes were not strongly biased in relation to HT. Height was significantly correlated with waist in males (r = .34, *p* < .001), but not in females, and not with either skinfold in either sex.

### Statistics

Conditional growth velocity is an outcome widely used to take into account the fact that size at any given time is correlated with size at previous time-points [21]. It indexes how much an individual has changed over time, taking into account this tendency for individuals to track. In each individual, conditional growth is quantified as the regression residual, from the regression of follow-up size on baseline size. These residuals are uncorrelated with prior measures of size. In our approach, we adjusted size at follow up (e.g., 6 months, or 12 months) for size at all available previous time-points, using sex-specific regression models. In other words, conditional weight gain represented the deviation during the preceding growth interval from the weight predicted by birth weight and other prior weights. The same approach was used to assess conditional length gain from length data.

For both weight and length/height, size at birth and conditional growth velocity were categorized into tertiles. Sex-specific analyses were then conducted, comparing tertiles 2 and 3 against tertile 1 for each sex. This approach was adopted because in many studies in industrialized populations, rapid weight gain has been associated with later obesity, and slow early weight gain has been suggested to reduce this risk and benefit cardiovascular health [4,8,9]. The sample size of 450 meant that tertiles had approximately 70 individuals of each sex, allowing us to detect a .5 standard deviation difference between upper and lower tertiles with 80% power, *p* < .5. The magnitude of the effect was assessed using Scheffe's post-hoc test. The mean difference and its 95% confidence intervals were given.

Correlations between conditional weight and conditional height were calculated for each of the 6, 12, and 48 time-points. General linear models were used to adjust the differences in body composition outcomes between conditional weight gain tertiles 2 and 3 and tertile 1 for conditional height gain as a continuous variable in the same period. The same approach was used to adjust differences between conditional height gain tertiles and conditional weight gain in the same period.

## Results

A comparison of descriptive characteristics of those in the subsample against those in the whole cohort is given in a supplementary online table. There were no significant differences between the subsample and the cohort except for birth weight z-score in males, which was .14 z-scores higher in the subsample, and weight at 1 year in both sexes, which was greater in the subsample. Although significant, the magnitudes of these effects are small, and unlikely to introduce bias into our findings. Table 1 presents anthropometry and body composition at 14 years. Males and females had similar BMI and BMI z-score. However, males were significantly taller and heavier, and had greater waist girth, LM, and LMI, while females had greater skinfolds, FM, and FMI.

### Associations between weight gain and later anthropometry

Table 2 shows that compared to the lowest birth weight tertile, the highest tertile had significantly greater adolescent height, though more so in males (Δ = 6.7 cm) than females (Δ = 4.2 cm). Those with highest conditional weights at 6 months showed a similar increment in height, again greater in magnitude in males (Δ = 7.4 cm) than females (Δ = 3.2 cm). For conditional weight gain at 12 months, the increment of being in the highest tertile was again higher in males (Δ = 5.3 cm) than females (Δ = 2.9 cm). However, for conditional weight at 48 months, the height increment for the highest tertile was the same in males and females (Δ = 6 cm).

Table 2 also shows that the largest tertile of birth weight did not have greater waist girth or skinfolds than the smallest birth weight tertile in either sex. However, those in the top conditional weight tertile at 6 months had greater waist girth and subscapular skinfold in females but not males. In contrast, boys in the top tertile at 12 months had greater waist and triceps skinfold, but this association was not observed among girls. Conditional weight at 48 months was associated with greater waist girth and both skinfolds in both sexes, and the magnitude of these effects was relatively similar between the sexes.

### Associations between weight gain and body composition outcomes

Table 3 presents similar data for body composition outcomes. There was a dose response association between birth weight tertile and LM in males but not females. The heaviest birth weight tertile also had greater FM in males, but not greater FMI, and no such association was apparent in females. At 6 months, the top tertile of conditional weight had greater LM in males, and greater LM, LMI, and FM in females. The LM increment was larger for males than females, whereas that for FM was larger in females. In the second 6 months, the top tertile of weight gain had greater LM and FM in males, and greater LM in females. The LM increment was larger for males than females, whereas that for FM was similar between the sexes. At 48 months, the top weight gain tertile had greater values than the bottom tertile for each of LM, LMI, FM, and FMI in males, but only for LM in females. Increments were larger for LM in males, but were similar between the sexes for FM. Thus, prior to one year, the only component of fat or lean mass that was increased beyond the equivalent effect on height was FMI in females, through rapid weight gain in the first six months of life.

### Associations between gain in length/height and body composition outcomes

Table 4 presents body composition outcomes according to the rate of length gain in fetal life, and conditional length/height at 6, 12, and 48 months. At birth, the longest tertile had greater adolescent LM in both males and females. In the first 6 months of life, those gaining length fastest had greater LM in both sexes, and greater FM and FMI in males. For females, there was also a dose-response association across the conditional height tertiles and lean mass. There was no difference in outcomes between groups of length gain in the second 6 months of life. Those in the top tertile of conditional height at 48 months had greater LM in both sexes, and greater LMI, FM, and FMI in males. Prior to one year, therefore, the only effect of rapid length gain apparent beyond effects on height was an increase in adiposity (fat mass and fat mass index) in males belonging to the top tertile of conditional length at 6 months.

### Comparisons across periods

**Figure 1:**
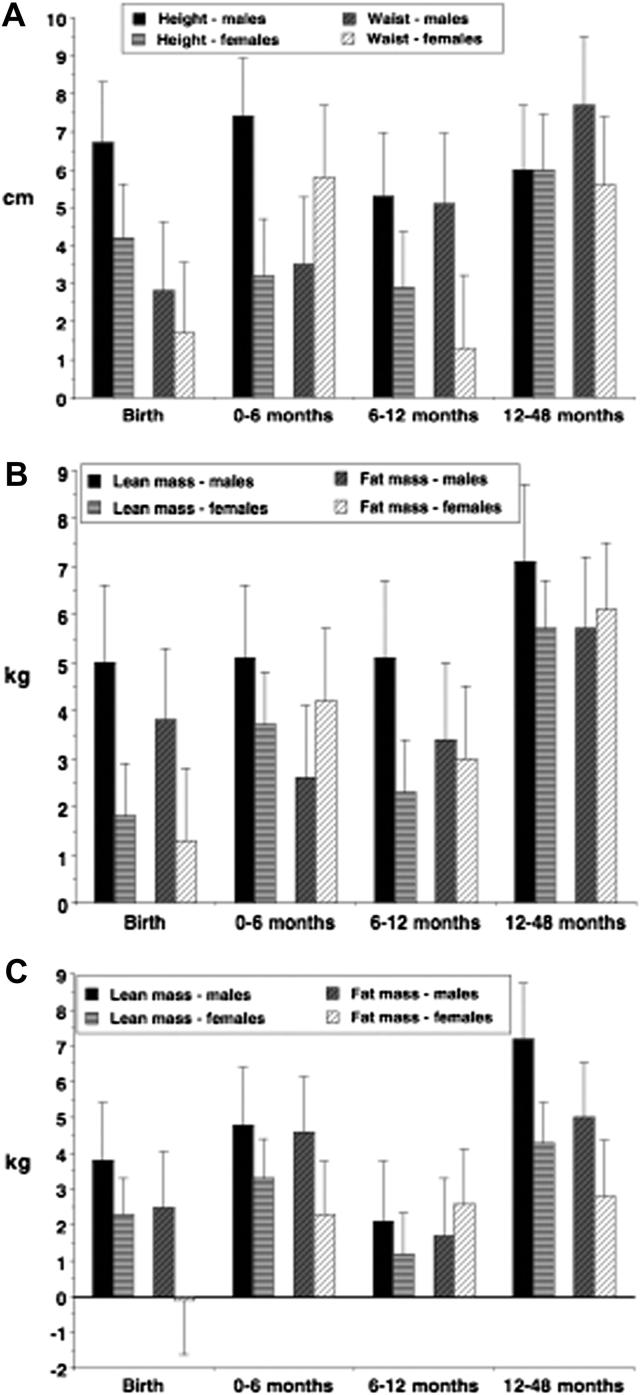
Each plot shows on the y-axis the magnitude of effect (expressed as Scheffe post-hoc coefficient from general linear model, with standard error) of being in the third versus the first growth tertile, in relation to different periods and measures of growth on the x-axis. (A) Height and waist circumference in cm (y-axis) in relation to birth weight or conditional weight gain over 3 periods (x-axis). (B) Lean mass and fat mass in kg (y-axis) in relation to birth weight or conditional weight gain over three periods. (C) Lean mass and fat mass in kg (y-axis) in relation to birth length or conditional length gain over three periods (x-axis).

The magnitude of effect of being in the fastest growth tertile, relative to the slowest tertile, is summarized in Figure 1 for different outcomes, stratified by different growth periods and exposures (weight or length gain). Figure 1A illustrates the magnitude of effect of being in the fastest compared to the slowest weight gain tertile for the outcomes height and waist girth, according to sex. The graph highlights a stronger effect of rapid weight gain on height in males than females up to one year of age, and stronger effect of rapid infant weight gain in the first 6 months on waist girth in females compared to males. Figure 1B illustrates the equivalent magnitude of effect for body composition outcomes (fat mass and lean mass). Rapid weight gain pays a larger dividend in LM in males than females at all time points, but especially during fetal life and from 6 to 12 months. From 6 months onwards, rapid weight gain generates relatively similar effects on later FM in the two sexes. However, being in the largest birth weight tertile generates a bigger effect in FM for males than females, whereas rapid weight gain in the first 6 months of post-natal life generates a bigger effect in FM for females than males.

The magnitude of effect of being in the fastest height growth tertile, relative to the slowest growth tertile, is summarized in Figure 1C for the outcomes fat mass and lean mass. Rapid length gain pays a larger dividend in LM in males than in females at all time points. Rapid length gain also pays a bigger dividend in FM in males than females during fetal life, early infancy, and early childhood. These sex differences are minimal in the second six months of post-natal life.

### Covariance between gains in weight and height

Conditional weight and length were correlated at 6 months (males: r = .62; females: r = .48), 12 months (males: r = .24; females: r = .30) and 48 months (males: r = .49; females: r = .55), *p* < .001 in all cases. Adjusting the associations between tertiles of conditional weight and adolescent body composition for the relevant conditional height made very little difference to the coefficients for the difference between the first and third tertile. In contrast, adjusting the associations between tertiles of conditional length and adolescent body composition for the relevant conditional weight reduced the magnitude of the difference between first and third tertiles, so that no differences remained significant except those for LM in males at 6 months. This effect also disappeared after adjustment for height, i.e., when LMI was the outcome.

## Discussion

Our data reveal multiple associations of conditional weights and heights—indexing rapid growth during different developmental periods—with body composition outcomes at 14 years of age in a Brazilian birth cohort. We chose to focus on rapid weight gain, using a tertile approach, as it has been suggested to be a risk factor for obesity in many studies of industrialized populations, both in infancy and early childhood [2,5,6]. We therefore compared rapid versus slow weight gain, recommended by some for long-term health [4,7–9], but we also repeated the main analyses for length gain. Our results add to previous work on anthropometric and skinfold outcomes in the same cohort [22] by providing accurate data on lean and masses, measured by stable isotopes.

In this population, rapid weight gain was always beneficial in terms of height and LM for males, but less consistently so for females. In terms of the adiposity indicators, birth weight was neutral except for an association with fat mass (but not FMI) in boys only. Weight gain after 12 months was particularly associated with fat mass, waist circumference, and skinfolds, in both sexes. Associations between weight gain up to 12 months and adiposity were weaker or nonexistent, and tended to be stronger for boys than girls.

Our data are consistent with previous work conducted in modernizing populations indicating that the primary consequence of rapid weight gain in early life is that both males and females become bigger, rather than fatter [10–13]. For each of birth weight and conditional weight at 6 and 12 months, there were no consistent associations with later adiposity once height was adjusted for, with the exception of weight gain 0–6 months and waist girth in females, and weight gain 0–6 months and FMI in males. In contrast, conditional weight at 48 months was associated with greater waist girth, skinfolds, and FMI in both sexes, though also with greater LMI. For females, compared to being in the slowest weight gain tertile, being in the fastest tertile between birth and 6 months was associated with an increment of +4.2 kg fat, whereas being in the fastest tertile between 12 months and 48 months was associated with an increment of +6.1 kg fat. Thus, rapid weight gain has stronger and more systematic associations with adiposity after the first year of postnatal life, suggesting that fetal life and infancy are less important periods of the life-course for obesity risk in this population. This fits with previous work in Brazil and other modernizing populations, suggesting that early gains in weight are beneficial for a wide variety of dimensions of human capital [23].

Length gain in early infancy showed a slightly different pattern to weight gain, with rapid length gain in males in the first 6 months of postnatal life associated with later FMI. Early gains in weight and length have previously been shown to have slightly different implications for later body composition [24]. Catch-up growth in length is associated with increased insulin sensitivity, which in turn has been associated with up-regulation of IGF1 and insulin receptors in muscle tissue [25]. These early hormonal adaptations may potentially elevate the risk of later excess weight gain if exposed to an insulinogenic diet [26,27]. Thus, although gains in length in early life may be very beneficial for height and LM, they may also elevate metabolic risk through the underlying hormonal adaptations, dependent on subsequent environmental exposures. Further work is required to explore such possible associations between early length gain patterns and diet composition. It is possible that the penalties of rapid length growth might be reduced by interventions in childhood rather than in infancy itself.

Conditional gains in weight and height in each period were correlated. In an earlier analysis of a larger sample of adolescents from the same cohort, conditional weight at 1 and 4 years tended to be associated with higher blood pressure, BMI, and skinfolds [22]. In marked contrast, rapid length/height gains tended to reduce BMI and skinfolds. These results only emerged when length/height gain and weight gain were adjusted for one another. In our analyses using deuterium-based body composition outcomes in a smaller subsample, similar findings were observed in terms of weight gain variables, but we did not reproduce the benefits reported in terms of length/height gain. In fact, adjusting the difference between fast and slow weight gain tertiles for length gain made little difference to the findings, whereas the equivalent adjustment of the difference between fast and slow length gain tertiles for weight gain reduced most of the significant differences to nonsignificance, suggesting that weight gain is broadly more important for later body composition than length gain.

The other main finding is that males and females show contrasting tissue “investment strategies” in early life. Rapid weight gain is more strongly associated with LM and height in males than in females, and with adiposity indices in females than in males. These early differences are consistent with previous data on tissue accretion patterns in early life, suggesting that a modest degree of sexual dimorphism in body composition is apparent in birth and infancy [28–31]. However, while the strategy for dimorphism is expressed primarily in adolescence, our data show that it is highly sensitive to weight gain in early infancy, but much less so to weight gain in early childhood.

The strengths of this study include the prospective nature of the anthropometric data, the use of conditional growth velocity to define independent indices of weight and length gain in successive developmental periods, and the use of an accurate stable isotope methodology to measure body composition. The limitations include the lack of data on actual tissue accretion patterns in early life, the lack of anthropometric data at 2 years, and the sample size. More subtle effects of rapid weight gain could be detected if larger numbers of adolescents had been studied. We also lacked an accurate approach for assessing central adiposity. We conducted multiple comparisons, which might increase the likelihood of a chance finding; however, our results are essentially conservative, in failing to find associations of growth and later adiposity reported in industrialized populations [1–6]. A potential limitation is that we did not adjust for breast-feeding status in infancy. However, previous work in this cohort has shown no association between breast-feeding and obesity status at 11 years [32], a finding perhaps unsurprising given that the majority of the cohort were breast-fed until 3 months [33].

In conclusion, rapid weight gain in early life in this population is associated with greater height, and after taking this size effect into account, associations of weight gain with later body composition were only systematically apparent from the second year of life onwards. Males and females have different patterns of associations, with rapid infant weight gain benefitting later height and lean mass more in males, and fat mass more in females. These data suggest that associations of weight gain and obesity may develop primarily after infancy in this population. The fact that infant weight gain is implicated in the magnitude of sexual dimorphism in adolescence may offer a clue as to why this developmental period has a lower effect on obesity risk.

## Figures and Tables

**Table 1:** Description of age, anthropometry and body composition

	Males (n = 222)		Females (n = 203)		*p*
	Mean	SD	Mean	SD	
Age (years)	14.1	.3	14.1	.4	.4
Weight	60.1	13.7	55.0	11.4	<.0001
Height (cm)	167.7	8.3	158.9	6.9	<.0001
BMI (kg/m2)	21.3	4.0	21.7	4.3	.2
BMI z-score^a^	.4	1.2	.3	1.2	.7
Waist girth	72.3	8.9	69.3	6.7	<.0001
Triceps skinfold (mm)	11.3	7.0	17.0	6.6	<.0001
Subscaps skinfold (mm)	9.8	5.6	13.1	6.3	<.0001
Lean mass (kg)	40.8	8.1	37.0	5.4	<.0001
Lean mass index (kg/m2)	16.0	2.0	15.1	1.8	<.0001
Fat mass (kg)	10.1	7.7	14.1	7.1	<.0001
Fat mass index (kg/m2)	3.9	2.8	5.7	2.8	<.0001

a Using WHO criteria.

**Table 2:** Adolescent height and regional adiposity according to tertile of birth weight or conditional weight gain in different growth periods

		n	Height (cm)			Waist girth (cm)			Triceps (mm)			Subscapular (mm)		
			Mean	Δ	95% CI	Mean	Δ	95% CI	Mean	Δ	95% CI	Mean	Δ	95% CI
Boys	Tertile													
Birth weight	1	70	164.4			71.4			10.7			9.6		
	2	75	167.2	2.8	−.4, 6.0	71.1	−.3	−3.9, 3.3	10.0	−.7	−3.6, 2.1	9.3	−.4	−2.7, 1.9
	3	76	**171.1**	**6.7**	**3.5, 9.9**	74.1	2.8	−.9, 6.4	13.1	2.4	−.4, 5.2	10.5	.9	−1.4, 3.2
6 months	1	72	163.4			70.5			11.0			9.6		
	2	65	168.6	5.2	2.0, 8.4	72.1	1.6	−2.1, 5.3	11.1	.1	−2.8, 3.0	10.12.11	.5	−1.8, 2.8
	3	79	**170.8**	**7.4**	**4.4, 10.5**	74.1	3.5	−.0, 7.1	11.7	.7	−2.1, 3.5	9.8	.2	−2.0, 2.4
12 months	1	69	164.8			69.9			9.9			9.2		
	2	73	168.0	3.2	−.1, 6.5	72.0	2.1	−1.5, 5.8	10.7	.9	−2.0, 3.7	9.3	.1	−2.2, 2.4
	3	68	**170.1**	**5.3**	**1.9, 8.6**	**75.0**	**5.1**	**1.4, 8.8**	**13.5**	**3.6**	**.7, 6.5**	11.0	1.8	−.6, 4.1
48 months	1	65	164.5			68.3			9.7			8.6		
	2	67	167.1	2.5	−.9, 6.0	71.3	2.9	−.6, 6.5	10.6	.9	−2.1, 3.8	9.5	.9	−1.4, 3.2
	3	69	**170.5**	**6.0**	**2.6, 9.4**	**76.1**	**7.7**	**4.1, 11.3**	**13.1**	**3.3**	**.4, 6.2**	**10.8**	**2.3**	**0.0, 4.6**
Girls	Tertile													
Birth weight	1	67	156.5			68.8			16.8			13.6		
	2	65	160.0	3.4	.6, 6.3	68.7	−.1	−3.8, 7.6	16.7	−0.0	−2.9, 2.8	12.5	−1.1	−3.9, 1.6
	3	68	**160.8**	**4.2**	**1.4, 7.1**	70.5	1.7	−2.0, 5.4	17.3	.5	−2.4, 3.4	13.4	−.2	−3.7, 1.8
6 months	1	63	156.7			66.4			15.6			11.4		
	2	73	160.4	3.6	.8, 6.5	69.3	2.8	−.7, 6.5	16.7	1.1	−1.7, 3.9	12.8	1.4	−1.2, 4.0
	3	59	**159.9**	**3.2**	**.2, 6.2**	**72.2**	**5.8**	**2.0, 9.6**	18.3	2.7	−.2, 5.6	15.4	4.1	1.3, 6.8
12 months	1	63	157.0			69.3			16.3			13.5		
	2	64	159.8	2.7	−.2, 5.7	68.0	−1.3	−5.2, 2.6	16.6	.2	−2.7, 3.2	12.1	−1.4	−4.2, 1.4
	3	63	160.0	2.9	−0.0, 5.9	70.6	1.3	−2.5, 5.2	17.9	1.6	−1.4, 4.5	14.2	.7	−2.1, 3.5
48 months	1	60	156.2			66.5			14.5			10.9		
	2	59	158.7	2.5	−.4, 5.5	68.4	1.9	−1.6, 5.5	17.2	2.7	−.2, 5.6	13.6	2.7	−.1, 5.4
	3	59	**162.2**	**6.0**	**3.1, 8.9**	**72.1**	**5.6**	**2.0, 9.1**	**18.5**	**4.1**	**1.2, 7.0**	**15.3**	**4.3**	**1.6, 7.1**

Values in bold text are significant, *p* < .05.

**Table 3:** Adolescent body composition according to tertile of birth weight or conditional weight gain in different growth periods

		n	Lean mass (kg)			Lean mass index (kg/m2)			Fat mass (kg)			Fat mass index (kg/m2)		
			Mean	Δ	95% CI	Mean	Δ	95% CI	Mean	Δ	95% CI	Mean	Δ	95% CI
Boys	Tertile													
Birth weight	1	75	38.0			15.5			9.0			3.7		
	2	77	**41.2**	**3.2**	**.1, 6.4**	16.2	.7	−.1, 1.5	8.8	−.2	−3.2, 2.8	3.5	−.2	−1.3, .9
	3	77	**43.0**	**5.0**	**1.8, 8.1**	16.1	.6	−.1, 1.4	**12.5**	**3.8**	**.6, 6.6**	4.7	1.0	−.1, 2.1
6 months	1	76	38.2			15.7			8.9			3.7		
	2	66	40.9	2.6	−.6, 5.9	15.8	.1	−.7, .9	9.7	.8	−2.4, 3.9	3.8	.1	−1.1, 1.3
	3	82	**43.3**	**5.1**	**2.0, 8.1**	16.3	.6	−.2, 1.3	11.6	2.6	−.4, 5.6	4.3	.6	−.5, 1.7
12 months	1	72	38.4			15.6			8.9			3.6		
	2	75	41.0	2.6	−.6, 5.8	16.0	.3	−.4, 1.1	9.4	.5	−2.6, 3.6	3.7	.1	−1.0, 1.3
	3	71	**43.5**	**5.1**	**1.8, 8.3**	16.4	.8	−0.0, 1.6	**12.3**	**3.4**	**.3, 6.6**	4.5	.9	−.2, 2.1
48 months	1	67	37.2			15.4			7.2			3.0		
	2	70	40.2	3.0	−.2, 6.1	15.9	.5	−.3, 1.3	9.7	2.4	−.6, 5.5	3.8	.8	−.4, 2.0
	3	71	**44.3**	**7.1**	**4.0, 10.3**	**16.6**	**1.2**	**.4, 2.0**	**12.9**	**5.7**	**2.6, 8.8**	**4.8**	**1.8**	**.7, 3.0**
Girls	Tertile													
Birth weight	1	70	36.1			15.1			13.9			5.8		
	2	67	37.0	.8	−1.4, 3.0	14.9	−.3	−1.0, .5	13.3	−.6	−3.6, 2.4	5.3	−.4	−1.6, .8
	3	70	38.0	1.8	−.4, 4.0	15.1	0.0	−.8, .7	15.2	1.3	−1.7, 4.3	6	.2	−.9, 1.4
6 months	1	66	35.6			14.9			12.3			5.1		
	2	76	36.6	.9	−1.2, 3.1	14.7	−.2	−.9, 1.6	13.9	1.5	−1.4, 4.4	5.6	.5	−.7, 1.6
	3	60	**39.3**	**3.7**	**1.4, 5.9**	**15.6**	**.7**	−**0.0, 1.5**	**16.5**	**4.2**	**1.1, 7.2**	**6.6**	**1.6**	**.3, 2.8**
12 months	1	66	35.9			15.0			13.1			5.4		
	2	65	36.9	1.1	−1.2, 3.3	15.0	−.1	−.8, .7	13.4	.3	−2.7, 3.4	5.4	−0.0	−1.2, 1.2
	3	66	**38.2**	**2.3**	**.2, 4.6**	15.3	.2	−1.1, .5	16.1	3.0	−.1, 6.0	6.4	1.0	−.2, 2.2
48 months	1	63	34.4			14.7			11.2			4.8		
	2	60	36.2	1.8	−.3, 4.0	14.9	.2	−.6, .7	13.9	2.7	−.3, 5.6	5.7	.9	−.3, 2.1
	3	62	**40.1**	**5.7**	**3.6, 7.8**	**15.5**	**.8**	**.1, 1.6**	**17.3**	**6.1**	**3.2, 9.0**	**6.6**	**1.8**	**.6, 3.0**

Values in bold text are significant, *p* < .05.

**Table 4:** Adolescent body composition according to tertile of birth length or conditional length gain in different growth periods

		n	Lean mass (kg)			Lean mass index (kg/m2)			Fat mass (kg)			Fat mass index (kg/m2)		
			Mean	Δ	95% CI	Mean	Δ	95% CI	Mean	Δ	95% CI	Mean	Δ	95% CI
Boys	Tertile													
Birth length	1	76	39.2			15.9			9.3			3.8		
	2	83	40.2	1.0	−2.1, 4.1	15.9	−0.0	−.8, .7	9.4	.1	−2.8, 3.1	3.7	−0.0	−1.1, 1.1
	3	70	**43.1**	**3.8**	**.5, 7.0**	16.1	.2	−.6, 1.0	11.8	2.5	−.6, 5.6	4.4	.6	−.6, 1.8
6 months	1	72	38.6			16.0			7.7			3.2		
	2	78	40.6	2.0	−1.1, 5.2	15.9	−.1	−.9, .7	10.2	2.5	−.5, 5.5	4.0	.8	−.3, 2.0
	3	74	**43.3**	**4.8**	**1.6, 8.0**	16.1	.1	−.7, .9	**12.3**	**4.6**	**1.6, 7.7**	4.5	**1.3**	**.2, 2.5**
12 months	1	75	40.1			16.0			10.0			4.0		
	2	76	40.7	.6	−2.6, 3.9	16.0	−0.0	−.8, .7	9.1	−.9	−4.0, 2.2	3.6	−.4	−1.6, .7
	3	67	42.7	2.1	−1.2, 5.5	16.0	−0.0	−.8, .8	11.6	1.7	−1.5, 4.9	4.3	.3	−.9, 1.5
48 months	1	70	37.5			15.6			8.0			3.3		
	2	68	39.7	2.2	−.9, 5.3	15.7	0.0	−.7, .9	9.0	1.0	−2.1, 4.1	3.6	.3	−1.5, .8
	3	70	**44.7**	**7.2**	**4.1, 10.3**	**15.2**	**.4**	**0.0, 1.6**	**13.0**	**5.0**	**1.9, 8.1**	**4.8**	**1.4**	**.3, 2.6**
Girls	Tertile													
Birth length	1	63	36.0			15.9			14.4			6.0		
	2	76	36.6	.6	−1.7, 2.9	15.0	−.1	−.9, .7	14.0	−.4	−1.5, 2.7	5.7	−.3	−1.5, 1.0
	3	66	**38.3**	**2.3**	**.1, 4.4**	15.0	−.1	−.8, .7	14.3	−.1	−3.1, 2.9	5.6	−.4	−1.6, .8
6 months	1	68	35.0			14.8			12.5			5.3		
	2	62	37.8	2.7	.5, 5.0	15.0	.4	−.4, 1.1	15.5	3.0	−.1, 6.0	6.2	.9	−.3, 2.1
	3	70	**38.3**	**3.3**	**1.1, 5.5**	15.4	−.2	−.4, .6	14.8	2.3	−.7, 5.2	4.5	.4	−.8, 1.5
12 months	1	63	35.9			15.2			12.4			5.2		
	2	70	38.0	2.1	−.3, 4.4	15.4	.2	−.6, 1.0	15.5	3.1	−0.0, 6.2	6.2	1.1	−.2, .1
	3	62	37.1	1.2	−1.1, 3.5	14.7	−.5	−1.3, .3	15.0	2.6	−.4, 5.7	6.0	.8	−.4, .2
48 months	1	58	35.2			15.2			13.3			5.7		
	2	65	36.0	.8	−1.4, 3.1	14.7	−.5	−1.3, .3	13.3	−.1	−3.2, 3.0	5.4	−.3	−1.5, .9
	3	60	**39.5**	**4.3**	**2.1, 6.6**	15.2	−0.0	−.8, .8	16.1	2.8	−.3, 5.9	6.1	.4	−.8, 1.7

Values in bold text are significant, *p* < .05.
