# Supplementary material for: Associations of Intrauterine and Postnatal Weight and Length Gains With Adolescent Body Composition: Prospective Birth Cohort Study From Brazil
Source: J Adolesc Health. 2012 Dec;51(6):S58–64. doi: 10.1016/j.jadohealth.2012.08.013 (PMC3508414; doi:10.1016/j.jadohealth.2012.08.013)
Supplement: Supplementary Online Table [file mmc1.docx]

**Supplementary online Table 1. Comparison of subsample with whole cohort**

Males Females

No (n = 1785) Yes (n = 222) No (n = 1896) Yes ( n = 203)

Mean SD Mean SD Mean SD Mean SD

Early life

Birth weight z-score -0.30 1.19 -0.16 * 1.14 -0.33 1.19 -0.23 1.24

Weight 1 year ^1^  9.7 1.5 10.2 * 1.1 9.2 * 1.2 9.5 1.2

Weight 4 years ^1^ 18.0 3.6 18.4 3.0 17.5 3.3 17.9 3.2

Adolescence

Weight (kg) 60.0 137. 60.1 13.7 54.8 11.0 55.0 11.4

Height (cm) 167.1 8.0 167.7 8.3 159.2 6.2 158.9 6.9

BMI (kg/m^2^) 21.4 4.0 21.3 4.0 21.6 3.9 21.7 4.3

Waist girth (cm) 72.4 8.9 72.2 8.9 68.9 8.3 69.3 8.7

Triceps (mm) 11.0 7.2 11.3 7.0 16.9 6.9 17.0 6.7

Subscapular (mm) 9.6 5.8 9.8 5.6 13.0 6.5 13.1 6.3

^1^ Sample size for those not followed up: 425 males and 409 females at 1 year; 340 males and 325 females at 4 years

* p<0.05
